# Does shoe heel design influence ground reaction forces and knee moments during maximum lunges in elite and intermediate badminton players?

**DOI:** 10.1371/journal.pone.0174604

**Published:** 2017-03-23

**Authors:** Wing-Kai Lam, Jaejin Ryue, Ki-Kwang Lee, Sang-Kyoon Park, Jason Tak-Man Cheung, Jiseon Ryu

**Affiliations:** 1 Li Ning Sports Science Research Center, Beijing, China; 2 Department of Kinesiology, Shenyang Sports Institute, Shenyang, China; 3 Biomechanics & Sport Engineering Laboratory, Kookmin University, Seoul, Korea; 4 Motion Innovation Centre, Korea National Sport University, Seoul, Korea; University e-Campus, ITALY

## Abstract

**Background:**

Lunge is one frequently executed movement in badminton and involves a unique sagittal footstrike angle of more than 40 degrees at initial ground contact compared with other manoeuvres. This study examined if the shoe heel curvature design of a badminton shoe would influence shoe-ground kinematics, ground reaction forces, and knee moments during lunge.

**Methods:**

Eleven elite and fifteen intermediate players performed five left-forward maximum lunge trials with Rounded Heel Shoe (RHS), Flattened Heel Shoe (FHS), and Standard Heel Shoes (SHS). Shoe-ground kinematics, ground reaction forces, and knee moments were measured by using synchronized force platform and motion analysis system. A 2 (Group) x 3 (Shoe) ANOVA with repeated measures was performed to determine the effects of different shoes and different playing levels, as well as the interaction of two factors on all variables.

**Results:**

Shoe effect indicated that players demonstrated lower maximum vertical loading rate in RHS than the other two shoes (*P* < 0.05). Group effect revealed that elite players exhibited larger footstrike angle, faster approaching speed, lower peak horizontal force and horizontal loading rates but higher vertical loading rates and larger peak knee flexion and extension moments (*P* < 0.05). Analysis of Interactions of Group x Shoe for maximum and mean vertical loading rates (*P* < 0.05) indicated that elite players exhibited lower left maximum and mean vertical loading rates in RHS compared to FHS (*P* < 0.01), while the intermediate group did not show any Shoe effect on vertical loading rates.

**Conclusions:**

These findings indicate that shoe heel curvature would play some role in altering ground reaction force impact during badminton lunge. The differences in impact loads and knee moments between elite and intermediate players may be useful in optimizing footwear design and training strategy to minimize the potential risks for impact related injuries in badminton.

## Introduction

Badminton is an intense sport that requires various fast and sudden movements [1–5]. Lunge is one of the most frequently executed footwork drills in badminton and it accounts for 15% of the total number of movements during a single game[2]. While the players expose to rapid and repetitive lunges that involve strenuous impact during heel contact phase, the recurrent sudden go-and-back task places harmful loads on the lower extremities of players that are suggested to cause high incidence of lower extremities injuries [6–8]. Therefore, appropriate footwear with optimal shock attenuation is needed to reduce the impact loads on the lower extremities during lunge movements.

Inappropriate shoe that fails to attenuate excessive shock on the human body has been identified as one risk factor for acute or fatigue overstress injuries [9–11]. Appropriate performance footwear should effectively attenuate impact forces generated during various impact activities[12]. Cushioning performance of footwear has largely been investigated in walking, running, and basketball landing movements [13–15] with only very few studies focused on badminton [7, 16]. In Hong et al. (2014) and Hu et al. (2015) studies, players were asked to perform badminton lunges in different directions while wearing different brands of shoes, but both of them failed to determine significant shoe differences on any ground reaction force and plantar pressure variables [7,16]. The different brands used in their studies might increase the number of confounding factors to overshadow the shoe effect. Ideally, shoes with isolated functional features should be tested in order to establish valuable guideline for footwear development [17].

In badminton, players often performed powerful and long-distance lunges having more than 40 degrees of the sagittal footstrike angle [7,16,18,19], which is much larger than heel-toe running having about 20 degrees[11]. Due to this unique sagittal footstrike angle and the location of initial contact, changes in heel curvature design of a badminton shoe might alter impact attenuation during lunge movements. Biomechanics knowledge about the effect of shoe heel design can assist footwear manufacturers to optimize shoe heel design, which aligns with badminton specific landing angle and aims for better cushioning performance to potentially reduce the risk of impact related injuries.

Furthermore, the footwear effect on impact forces could be participant-dependent [20–22]. Players of different levels would perform lunging with unique techniques and patterns, which might have caused to different biomechanical outcomes and thereby required adequate footwear design to reduce the harmful mechanical responses during lunges. Therefore, the present study aimed to examine the effect of shoe heel designs on shoe-ground kinematics, ground reaction force (GRF) and knee joint loading during lunge in different skill levels of badminton players. It was hypothesized that 1) rounded heel shoes might alter shoe-ground kinematics and lower GRF impact and knee joint loading during lunge compared with flatted heel shoes and 2) elite players might elicit larger sagittal footstrike angle, greater GRF and knee joint moments during lunge compared with less skilled players.

## Materials and methods

### Participants

Eleven male elite badminton players [mean age 20.6 (SD = 0.7, range = 20 to 22) years; height 176.0 (SD = 6.0) cm; mass70.9 (SD = 5.9) kg; year of badminton playing 8.4 (SD = 1.4) years; maximum lunge distance 149.2 (SD = 9.6), eight out of eleven players achieved Korean collegiate championship titles during the year of data collection] and fifteen male intermediate badminton players [mean age 21.4 (SD = 1.6, range = 20 to 25) years; height 176.0 (SD = 6.0) cm; mass66.9 (SD = 5.7) kg; year of badminton playing 3.2 (SD = 1.0) years; left maximum lunge distance 144.6 (SD = 9.0) cm] were recruited for this study. A priori power analysis was performed to determine appropriate sample sizes. Effect sizes were calculated from previous research with methods that closely resembled this study [19, 23]. Based on an alpha of 0.05 and 80% power, 11 subjects per group were recruited for this study. In addition, previous studies investigating badminton lunges between elite and intermediate players have reported their findings based on eight participants with significant group mean differences of lower limb biomechanics variables [24]. For elite participants, they were practicing with national team training at Korean National Sports University and other universities in Seoul. For intermediate participants, they were the convenient subjects who played in badminton club at Kookmin University. Independent *t*-test revealed no significant difference of age, height, mass, and lunge distance between groups (*P* > 0.05). All players were right-handed and were free from any lower extremity injuries for at least six months prior to the start of the study. Written consent was obtained from the participants and the testing procedure was approved by the Kookmin University Institutional Review Board.

### Footwear conditions

**Fig 1:**
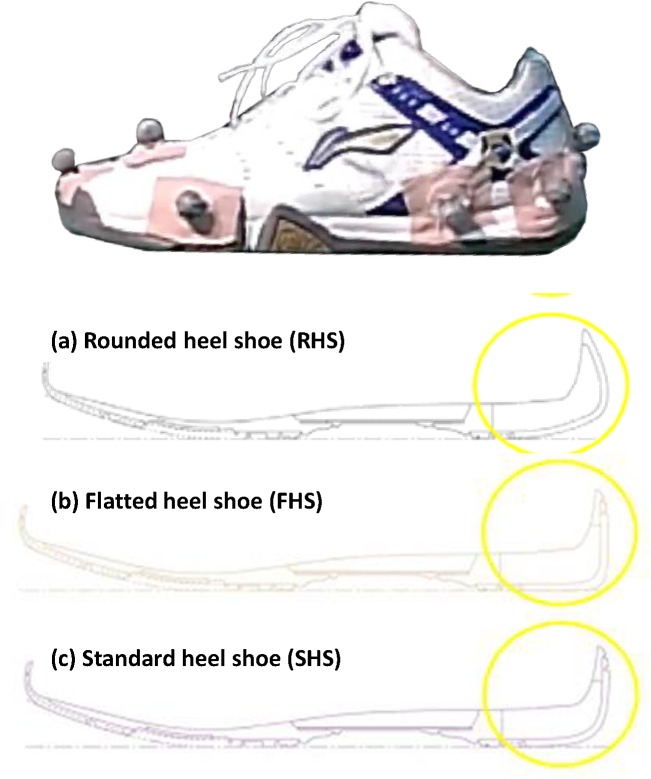
Footwear conditions. (a) Rounded Heel Shoe (RHS), (b) Flattened Heel Shoe (FHS), and (c) Standard Heel Shoe (SHS).

Three identical pairs of badminton shoes (Fig 1, Li Ning SAGA, Beijing, China) with different modifications of heel shape: Rounded Heel Shoe (RHS), Flattened Heel Shoe (FHS), and Standard Heel Shoe (SHS) were built. The SHS shoe condition was unmodified from its original specifications available in the market. The RHS was built with the 5-mm extension at the tip of the posterior heel compared to the SHS, which was selected based on the reference of existing available professional badminton shoe models. The FHS was built with flattened edges at the posterior heel.

### Apparatus and tasks

**Fig 2:**
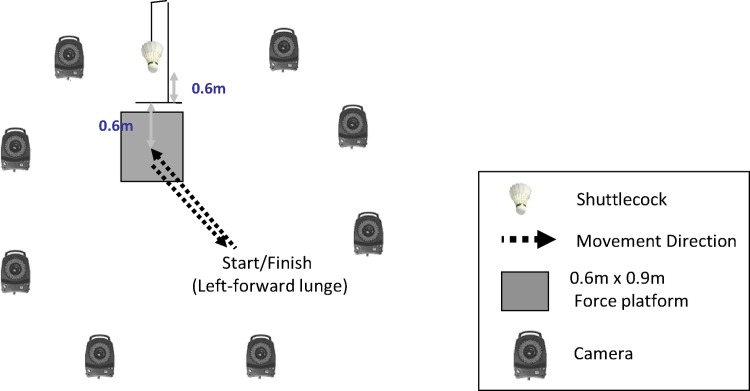
Experimental setup.

All players performed five maximum lunge trials in left-forward direction wearing each of the experimental shoes in random order (generated at www.random.org), as the left-forward lunge was recommended as one key lunge direction in badminton lunge research [7]. Professional badminton mat (Li Ning CP55 Premium Court Mat) was glued to the top of the force plate (Advanced Mechanical Technology Inc, Watertown, USA) and its surrounding surface to replicate realistic badminton shoe and ground interfaces. The force plate and an eight-camera infrared motion analysis system (Oxford Metrics Ltd, Oxford, UK) were synchronized to collect the GRF and kinematic information of the players during lunge at a sampling frequency of 1000Hz and 200Hz, respectively. Prior to data acquisition, both static and dynamic calibrations were performed to determine the position and orientation of the capturing volume in order to minimize the lens distortion of each camera (Fig 2). The cameras were aligned in a circular fashion to allow capturing of all reflective markers during lunge landing. The force plate was placed at a45-degree position relative to the left-forward direction of the starting positions. A shuttlecock was suspended at a height of 0.6 m with a string, which was 0.6 m from the center of the force plate along the respective lunging directions [19].

### Procedure

After completing the anthropometric measurements, players wore a new pair of standard socks and performed a 10-minute warm-up including stretching and jogging with their own badminton shoes. A total of 21 reflective markers (diameter 14 mm) were attached over the anatomical landmarks [19]: four pelvis markers (left and right ASIS and PSIS), medial and lateral epicondyles of femur, medial and lateral malleolus, two calcaneus markers (posterior upper, posterior lower of calcaneus of the shoe), three shoe tracking markers (medial side of first metatarsal head, upper side of second metatarsal head, and lateral side of fifth metatarsal head), and two four-marker rigid clusters were affixed to the thigh and leg segments. The markers on the medial and lateral malleolus and femoral epicondyles were used for the static trial and were removed during the dynamic trials.

Prior to actual data collection, participants familiarized themselves with lunging and tightened the lacings according to their individual preference for a badminton game. Three lunge trials with maximum-effort were used to determine the individual maximum lunge distance across shoe conditions. During each lunge step, players holding their badminton racket (Li Ning Ultra Carbon 9000) were instructed to initiate the lunge movement from the start position along the left-forward lunge direction. Players were instructed to extend their right knee and land on the force platform whilst hitting the target shuttlecock with the standard racket in order to simulate the actual frontcourt shuttle-drop situation in a badminton game. After hitting the shuttlecock, players were required to return to the start position as fast as possible. Successful trial consisted of correct foot placement at the lunge start line, contact of the lunging (front) leg with the center of the force plate, hit the shuttlecock and recovery to the starting point within three seconds[2,25]. The trial was discarded if the obvious slippage and discontinuity of movement was present. Five successful lunge trials were obtained for each shoe condition and for both lunge directions. Shoe conditions and lunge directions were randomized across participants. To minimize the effect of fatigue, 1.5-minute and 10-minute resting periods were mandatory between trials and between shoe conditions, respectively, as specified based on the previous badminton lunge study[7] and the participants’ feedback of our pilot testing.

### Data analysis

A spline interpolation was performed to rectify minor missing motion capture data using three frames of data before and after the missing data [26]. A fourth-order Butterworth bidirectional filter with a cut-off frequency of 12 Hz was used to smooth the kinematics data [19,27]. The kinetic data was filtered using a fourth order Butterworth filter at 100 Hz [2,27]. All marker trajectories and GRF signals were identified manually using Vicon Clinical Manager Software (Oxford Metrics Ltd, Oxford, UK) and then imported into Visual3D software (C-Motion Inc., Ontario, Canada) for definition of body segments and calculation of knee joint variables.

The contact phase of the lunge step was identified as the period from initial heel contact of the landing foot to toe-off, as determined by the force plate. The instance of heel contact and toe-off were defined when the vertical GRF first exceeded 10N (heel contact) and reduced to 10N (toe-off). Peak vertical and resultant horizontal forces and the respective loading rates were calculated for footwear cushioning performance evaluation [7,28,29]. The mean loading rate of impact GRF was calculated using the approach described in previous footwear studies [19] by obtaining the force slope during the loading period in which the force increased from 20% to 90% of the impact force peak magnitude. The maximum loading rate was defined as the maximum value reached by the force slopes during every 1% of the stance period from initial heel contact to impact force peak [7].

Knee joint angle was defined as the orientation of one distal segment (i.e., shank) relative to the proximal segment (i.e., thigh). Only knee flexion and extension moments were evaluated in the present study because the knee joint motion during lunge falls primarily in the sagittal plane, which contributes to the major knee biomechanical characteristics in badminton research [2,19,25,30]. Approaching time, total contact time, and sagittal footstrike angle were measured to provide the general movement characteristics for respective elite and intermediate groups. The GRF and knee kinetics variables were normalized by the individual subject’s body weight (BW) prior to further data processing [2].

All statistical analyses were performed using SPSS 21.0 (IBM Corp., Armonk, NY, USA). Being robust to moderate violations of normality observed in most of the data, a 2 (Group) x 3 (Shoe 3) repeated measures ANOVA was performed on each of all variables. To assess the reliability between the test trials for each condition, coefficient of variation (CV) was calculated on each of all variables. Bonferroni corrected post-hoc tests were performed to correct multiple measurements. Alpha was set at 0.05 for all analyzes. Greenhouse-Geisser’s epsilon adjustment was used in all cases when Mauchly’s test indicated that the sphericity assumption had been violated.

## Results

### Shoe-ground kinematics

Two-way repeated measures ANOVA did not determine any effect of Shoe and interaction between Group and Shoe for all movement characteristics variables (*P* > 0.05) (Table 1). Significant Group effect was found with the sagittal footstrike angle (*F*(1,24) = 43.31, *P* < 0.01). The post-hoc analysis indicated that compared to the intermediate players, elite players exhibited significantly larger sagittal footstrike angle during lunge (*P* < 0.01).

**Table 1:** ANOVA results on shoe-ground kinematics variables in Rounded Heel Shoe (RHS), Flattened Heel Shoe (FHS), and Standard Heel Shoe (SHS) by expertise.

Variable	Group	Shoe			Shoe effect			Group effect			Interaction		
		RHS	FHS	SHS	*P*	η^2^	*β*	*P*	η^2^	*β*	*P*	η^2^	*Β*
Approaching time (s)	E	0.82(0.30)	0.76(0.30)	0.74(0.28)	.59	.02	.13	.74	<.01	.06	.43	.04	.19
	I	0.73(0.41)	0.69(0.34)	0.78(0.40)									
Contact time (s)	E	0.71(0.14)	0.69(0.14)	0.69(0.12)	.17	.07	.38	.14	.09	.32	.17	<.01	.07
	I	0.78(0.10)	0.75(0.10)	0.75(0.10)									
Sagittal footstrike angle (deg)	E	44.58(5.06)	43.72(5.13)	43.71(4.43)	.39	.04	.21	**<.001**	.64	1.00	.98	<.01	.05
	I	31.93(5.84)	31.11(5.75)	30.82(5.59)									

(E = elite; I = intermediate; Significant *P*-values (*P* <.05) are shown in bold)

### Ground reaction force variables

**Fig 3:**
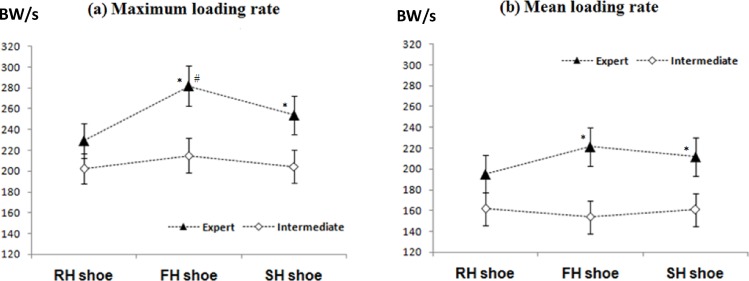
**Shoe x Group interaction for (a) maximum vertical loading rate and (b) mean vertical loading rate during left lunge direction.** *represents significant difference between RHS; #represents significant difference between SHS.

Interactions of Group x Shoe were found in maximum loading rate (*F*(2,48) = 4.93, *P* < 0.05) and mean loading rate (*F*(2,48) = 5.14, *P* < 0.05) of the vertical impact peak (Table 2). The simple main effect revealed that elite group showed a significant Shoe effect on maximum vertical loading rate (*F*(2,20) = 25.38, *P* < 0.01) and mean vertical loading rate (*F*(2,20) = 20.26, *P* < 0.01), while the intermediate group did not show any Shoe effect on the vertical loading rates (Fig 3). Simple main effect of Group revealed that elite players exhibited lower left maximum and mean vertical loading rates in RHS compared to FHS (*P* < 0.01) and SHS (*P* < 0.05) and lower maximum vertical loading rate in SHS than FHS (*P* < 0.05) (Fig 3)

**Table 2:** ANOVA results on ground reaction force variables in Rounded Heel Shoe (RHS), Flattened Heel Shoe (FHS), and Standard Heel Shoe (SHS) by expertise.

Variable	Group	Shoe			Shoe effect			Group effect			Interaction		
		RHS	FHS	SHS	*P*	η^2^	*β*	*P*	η^2^	*β*	*P*	η^2^	*Β*
Peak vertical impact force (BW)	E	2.18(0.34)	2.26(0.30)	2.30(0.29)	.54	.03	.15	.10	.11	.37	.64	.02	.12
	I	2.49(0.48)	2.46(0.34)	2.51(0.49)									
Peak resultant horizontal force (BW)	E	1.65(0.34)	1.71(0.32)	1.64(0.34)	.80	.01	.08	**<.001**	.53	1.00	.35	.04	.23
	I	2.47(0.45)	2.46(0.47)	2.51(0.47)									
Max vertical loading rate (BW/s)	E	229.2(55.9)	281.9(57.9)	254.2(47.4)	**<.001**	.36	1.00	**.047**	.16	.52	**.02**	.18	.72
	I	202.2(55.2)	214.9(68.5)	204.1(69.2)									
Mean vertical loading rate (BW/s)	E	195.3(43.2)	221.6(38.6)	211.6(33.0)	.18	.07	.35	**.04**	.16	.55	**.02**	.02	.80
	I	161.6(70.2)	153.9(73.5)	160.7(76.0)									
Max resultant horizontal loading rate (BW/s)	E	69.1(11.1)	76.9(10.9)	70.7(8.4)	.33	.05	.24	**<.001**	.65	1.00	.61	.61	.13
	I	129.3(30.1)	130.6(27.6)	128.7(31.6)									
Mean resultant horizontal loading rate (BW/s)	E	30.0(11.3)	31.8(12.6)	30.9(11.0)	.93	<.01	.06	**<.001**	.51	1.00	.75	.75	.09
	I	62.5(21.5)	61.4(17.9)	63.2(19.9)									

(E = elite; I = intermediate; Significant *P*-values (*P* <.05) are shown in bold)

Significant Group effect indicated that compared to the elite players, the intermediate players demonstrated significantly higher peak horizontal force (*P*< 0.01), maximum and mean horizontal loading rate (*P*< 0.01) while lower maximum and mean vertical loading rates (*P* < 0.01) (Table 2). In addition, post-hoc analysis of Shoe effect indicated that players wearing RHS demonstrated lower maximum vertical loading rate compared to wearing FHS (*P*< 0.01) and SHS (*P* < 0.05) (Table 2).

### Knee kinetic variables

Two-way repeated measures ANOVA did not determine any effect of Shoe and interaction between Group and Shoe for knee moment variables (*P*> 0.01) (Table 3). Significant Group effect indicated that compared to intermediate players, elite players had significantly higher peak knee extension moment (*P*< 0.01) and peak knee flexion moment (*P*< 0.01) during lunge (Table 3).

**Table 3:** ANOVA results on knee kinetics variables in Rounded Heel Shoe (RHS), Flattened Heel Shoe (FHS), and Standard Heel Shoe (SHS) by expertise.

		Shoe			Shoe effect			Group effect			Interaction		
Variable	Group	RHS	FHS	SHS	*P*	η^2^	*β*	*P*	η^2^	*β*	*P*	η^2^	*β*
Peak knee extension moment (Nm/BW)	E	1.27(0.55)	1.34(0.53)	1.20(0.64)	.35	.04	.23	**.01**	.24	.76	.30	.05	.25
	I	0.80(0.30)	0.81(0.31)	0.82(0.30)									
Peak knee flexion moment (Nm/BW)	E	0.88(0.26)	0.89(0.21)	0.90(0.30)	.74	<.01	.08	**<.001**	.43	.98	.61	.02	.11
	I	0.52(0.28)	0.50(0.37)	0.43(0.30)									

(E = elite; I = intermediate; Significant *P*-values (*P* <.05) are shown in bold)

### Coefficient of variation variables

Analysis of CV data revealed significant group effect on contact time, sagittal footstrike angle, peak vertical impact force, mean vertical loading rate, and max horizontal loading rate (*P* < 0.05), while no significant shoe effect or interaction effect was determined on any variables (*P* > 0.05) (Table 4). Compared to intermediate group, elite players had lower CV for contact time, sagittal footstrike angle, peak vertical impact force and mean loading rate (*P* < 0.05) but higher CV for maximum resultant horizontal loading rate (*P* < 0.05) (Table 4).

**Table 4:** Analysis of coefficient of variation (CV) of all variables in Rounded Heel Shoe (RHS), Flattened Heel Shoe (FHS), and Standard Heel Shoe (SHS) by expertise.

Variable (CV, %)	Group	Shoe			Shoe effect			Group effect			Interaction		
		RHS	FHS	SHS	*P*	η^2^	*β*	*P*	η^2^	*β*	*P*	η^2^	*β*
Approaching time	E	47.12(15.15)	33.04(10.20)	44.43(17.88)	.29	.05	.27	.81	<.01	.06	.13	.08	.42
	I	43.46(17.16)	44.00(13.23)	39.77(16.51)									
Contact time	E	4.67(2.15)	4.08(1.42)	4.85(2.00)	.71	.01	.10	**<.001**	.45	.99	.69	.02	.11
	I	10.92(6.90)	9.81(5.69)	9.19(4.30)									
Sagittal footstrike angle	E	6.18(3.32)	4.93(2.08)	5.59(1.94)	.38	<.01	.21	**<.01**	.29	.86	.76	.01	.09
	I	8.09(2.03)	7.63(2.95)	8.42(3.57)									
Peak vertical impact force	E	4.97(1.66)	3.79(1.63)	5.91(1.80)	,62	.02	.12	**<.01**	.31	.89	.69	.01	.09
	I	9.44(5.59)	9.34(5.67)	9.45(7.01)									
Peak resultant horizontal force	E	11.24(4.65)	7.77(3.19)	10.70(4.60)	.42	.04	.20	.52	.02	.10	.13	.08	.42
	I	9.20(3.50)	9.80(3.85)	8.91(4.04)									
Max vertical loading rate	E	10.89(4.52)	10.25(3.32)	12.84(4.94)	.73	.01	.10	.86	<.01	.05	.15	.08	.39
	I	11.10(4.53)	12.61(4.89)	10.88(3.35)									
Mean vertical loading rate	E	4.97(1.66)	3.79(1.63)	5.91(1.80)	.62	.02	.12	**<.01**	.31	.89	.69	.02	.11
	I	9.44(5.59)	9.34(5.67)	9.45(7.01)									
Max resultant horizontal loading rate	E	17.48(5.24)	19.09(7.65)	21.13(7.97)	.22	.06	.31	**.02**	.20	.64	.52	.03	.16
	I	13.40(6.24)	15.13(5.53)	14.48(7.28)									
Mean resultant horizontal loading rate	E	11.24(4.65)	7.77(3.95)	10.70 (4.60)	.42	.04	.20	.52	.02	.10	.13	.08	.42
	I	9.20(3.50)	9.80(3.85)	8.91(4.04)									
Peak knee extension moment (Nm/BW)	E	29.04(19.29)	18.99(11.64)	37.25(45.11)	.14	.09	.36	.78	<.01	.06	.26	.05	.24
	I	28.65(16.27)	24.40(14.69)	26.53(16.00)									
Peak knee flexion moment (Nm/BW)	E	28.38(9.53)	38.65(11.87)	28.07(13.31)	.27	.05	.26	.06	.15	.49	.05	.12	.56
	I	47.84(18.77)	39.48(21.24)	38.85(18.58)									

## Discussion

The present study examined the influence of shoe heel design (RHS, FHS versus SHS) on shoe-ground kinematics, external impact and knee loading during badminton lunge in elite and intermediate players. Results indicated that players wearing the RHS experienced lower maximum vertical impact loading rate during lunge, suggesting that the rounded heel structure enabled better shock attenuation in badminton lunges [7,12]. The current study also support the contention [31] that extreme rear of the heel region would alter the impact attenuation for lunging, while this area not normally associated with consistent high impact forces in other sports. However, no evidence would be found if the shoe heel design would influence knee loading variables during lunge step landing. It is possible that compared to knee loading and GRF magnitude variables, impact loading rate would be the critical differentiating variables for footwear evaluation and injury prediction in badminton lunge. The similar conclusion was suggested by the running meta-analysis [32], which showed that compared to healthy controls, subjects developing lower-limb stress fractures showed significantly higher maximum and average loading rates, but no significant difference in GRF peaks during running.

Although there was no statistically difference in maximum lunge distance between elite and intermediate players, differences in biomechanical performance exists. Compared to intermediate players, elite players were expected to have higher demands on lower extremities strength and efforts for their larger sagittal footstrike angle and faster approaching speed during lunge. The GRF results indicated that elite players were capable of effectively attenuating the heel landing impact for lower peak horizontal force and loading rate, but not for vertical loading rates. It suggested that the elite players might enable efficient joint coordination and ankle (or heel) rolling mechanics [33] and muscle damping[34]for absorbing horizontal impact loading whilst generating a more powerful and efficient lunge.

Additionally, the knee kinetic results suggested that the elite players elicited the higher efficiency of dealing with impacting forces and immediately move back with greater mechanical outputs productions [2] maximal lunge compared with the intermediate players. This partially supports the maximum dynamic hypothesis [35] that the muscular system of the lower limbs is designed to optimize dynamic output, which suggested that the elite players were expected to allow higher efficiency in dynamic output, possibly through kinetic and kinematic alterations to lunge movement. Future studies should explore the underlying muscle activation strategy in pre-contact and impact phases and determine the degree of self-organization using minimal number of movement components and electroencephalography [36–38] between elite and intermediate players in badminton lunge to aid coaches and physicians to optimize the training and rehabilitation protocols for better athletic performance and minimized injuries in badminton.

Interestingly, the performance of different shoe heel designs on the GRF loading responses of the two test groups was not consistent. Elite players had significant lower maximum and mean vertical loading rates in RHS than FHS and SHS, while intermediate players did not have any difference among shoe conditions (Fig 3). One plausible explanation is that intermediate players had larger movement variations (i.e. higher CV% in contact time, sagittal footstrike angle, peak vertical impact force and mean vertical loading rate) in execution of lunge, which might have hindered the actual shoe effect, whereas elite players would be more sensitive in responding to such a minimal change of shoe heel construction during lunge step landing. Another explanation would be the difference in sagittal footstrike angle in lunge movement and allow automaticity in muscle control strategies [39]. Based on the current knowledge indicating that running injury and performance are largely related to individual preference and footstrike style [34]. It is suggested that compared to the midfoot/forefoot strikes (with < 8° sagittal footstrike angle), runners with rearfoot strike landing (with > 8° sagittal footstrike angle) had obvious impact peak and 10 to 15% higher loading rate [40], which suggested that small amount of differences in sagittal footstrike might have caused different impact and loading responses. Future studies is necessary to investigate the effect of landing biomechanics, movement intensity, and muscular control on different heel structure designs for badminton lunge to provide further insights into the biomechanics of sports injuries.

When interpreting our results, it is important to consider several limitations in our study. First, although the test shoes were built with the identical sole thickness, the shoes with various curvature designs at the posterior aspect of the heel (Rounded, Flattened, Standard) might slightly influence the material thickness at the instant of initial contact. Computational study may help to simulate the impact characteristics incorporating material, shape and thickness. Second, only male participants were recruited and hence our findings may not be generalized to female badminton players. Third, only lunge movement was measured. Since other badminton movements such as smashing and high clear involve considerable vertical landing, interplay with gender and movement types would allow comprehensive information for heel shoe design in badminton.

## Conclusions

Shoe heel curvature design would play some roles in altering badminton lunge impact characteristics. The rounded heel construction was associated with lower impact loading rate during badminton lunge in both elite and intermediate players. Lunge movement of elite players was characterized with larger sagittal footstrike angle, smaller horizontal impact loading but larger vertical impact and knee moments, suggesting that elite and intermediate players might elicit distinct movements control strategies that need to be considered during biomechanics evaluation as well as designing athletic training and footwear suitable for different skilled players.
